# Polymer Nanomaterials in Biomedicine

**DOI:** 10.3390/ijms24087480

**Published:** 2023-04-19

**Authors:** Hyeonseok Yoon

**Affiliations:** 1 School of Polymer Science and Engineering, Chonnam National University, 77 Yongbong-ro, Buk-gu, Gwangju 61186, Republic of Korea; hyoon@chonnam.ac.kr; 2 Department of Polymer Engineering, Graduate School, Chonnam National University, 77 Yongbong-ro, Buk-gu, Gwangju 61186, Republic of Korea

Polymer nanomaterials have emerged as a promising class of materials within the field of biomedicine, owing to their unique physical, chemical, and biological properties. These materials have been extensively investigated for a wide range of biomedical applications, including drug delivery, tissue engineering, and diagnostic imaging. In this editorial, we review recent research articles about the use of polymer nanomaterials in biomedicine. More specifically, we examine the development of new nanomedicines in combination therapy for cancer treatment, the use of thermosensitive liquid suppositories for drug delivery, and the development of nanoparticulate photoluminescent probes for bioimaging. These articles highlight the potential of polymer nanomaterials in addressing some of the key challenges in biomedicine, such as the improvement of drug delivery, the enhancement of therapeutic efficacy, and the development of new diagnostic and imaging tools. As the field of polymer nanomaterials continues to advance, it is important to consider their safety and long-term effects. With careful designs and tests, polymer nanomaterials have the potential to revolutionize the field of biomedicine and improve patient outcomes.

Anna et al. [1] focused on the interaction between cationic pyridylphenylene dendrimers and anionic liposomes, studying how the composition of the dendrimers affects the structural changes in the membrane. The researchers discovered that dendrimers can form stable complexes with liposomes. The resulting changes in the membrane structure, for example, increase its permeability and disrupt the lipid bilayer. Moreover, the researchers discovered that the sizes and charges of these dendrimers have a significant impact on the degree of the structural changes in the liposome membrane. Overall, the study provided a helpful insight into the potential use of dendrimer–liposome complexes for drug delivery and other biomedical applications.

Kim et al. [2] developed a new nanomedicine based on polymersomes that could be used in combination therapy for cancer treatments. The nanomedicine contained both a chemotherapeutic drug and sonosensitizer, which could be activated by ultrasound to induce cell death in cancer cells. The researchers discovered that the polymersome-based nanomedicine was stable and biocompatible. The promising results of the in vitro and in vivo samples demonstrated significant tumor growth inhibition and prolonged survival in a mouse model. The study highlighted the potential of polymersome-based nanomedicines for the development of new cancer therapies.

Moreover, Maria et al. [3] developed a new thermosensitive liquid suppository containing metoprolol, which is a beta-blocker that is used for the treatment of cardiovascular diseases. The suppository was based on poly(lactide-co-glycolide) nanoparticles, which provided a sustained release of the drug and improved its bioavailability. The researchers discovered that the suppository exhibited thermosensitivity; thus, it could undergo a reversible sol-gel transition when exposed to body temperature, thereby providing a prolonged drug release and an enhanced therapeutic efficacy. The study report provided a comprehensive characterization of the suppository (including its stability, drug release profile, and cytotoxicity) and demonstrated its potential as a new drug delivery system for metoprolol.

Magdalena et al. [4] explored the effect of different topmost layers and types of bone morphogenetic protein-2 (BMP-2) immobilization on the response of mesenchymal stem cells (MSCs). The researchers investigated the effects of three different topmost layers (a cationic polymer, an anionic polymer, and a hydrophilic linker) and two different types of BMP-2 immobilization (physical adsorption and covalent bonding) on the behavior of these MSCs. The results of this study demonstrated that both the topmost layer and type of BMP-2 immobilization significantly affect the adhesion, proliferation, and differentiation of MSCs. The results suggested that the use of an anionic polymer and the covalent bonding of BMP-2 could enhance the osteogenic differentiation of MSCs, while the use of a cationic polymer and physical adsorption could enhance their cell adhesion and proliferation. The study report provided valuable insight into the design of biomaterials for bone tissue engineering and highlighted the importance of carefully selecting the topmost layer and type of BMP-2 immobilization for an optimal MSC response.

Lee et al. [5] studied the development and use of nanoparticulate photoluminescent probes for bioimaging applications. The study covered small molecule- and polymer-based probes, which were used to label and visualize cells, tissues, and organs in vivo and in vitro. The study highlighted the advantages and limitations of different nanoparticulate probes (such as their size, photophysical properties, and biocompatibility) and provided examples of their uses in various bioimaging techniques, including fluorescence microscopy, positron emission tomography, and magnetic resonance imaging. The researchers also discussed the challenges and future directions for the development of nanoparticulate probes for bioimaging, including the improvement of their sensitivity, specificity, and targeting capabilities. Overall, the study emphasized the importance of nanoparticulate probes in advancing our understanding of biological systems and developing new diagnostic and therapeutic tools.

The use of polymer nanomaterials in biomedicine is an exciting and rapidly growing field with a wide range of potential applications. The articles reviewed in this editorial highlight some of the latest developments and advancements within the field, including the development of new nanomedicines in combination therapy for cancer treatment, the use of thermosensitive liquid suppositories for drug delivery, and the development of nanoparticulate photoluminescent probes for bioimaging.

Furthermore, these studies demonstrate the versatility and potential of polymer nanomaterials in addressing some of the key challenges in biomedicine, such as the improvement of drug delivery, the enhancement of therapeutic efficacy, and the development of new diagnostic and imaging tools. The use of these materials can lead to better outcomes for patients; that is, disease management can be improved and survival rates can be increased. 
